# Development and comparison of novel bioluminescent mouse models of pancreatic neuroendocrine neoplasm metastasis

**DOI:** 10.1038/s41598-021-89866-1

**Published:** 2021-05-13

**Authors:** Courtney A. Kaemmer, Shaikamjad Umesalma, Chandra K. Maharjan, Devon L. Moose, Goutham Narla, Sarah L. Mott, Gideon K. D. Zamba, Patrick Breheny, Benjamin W. Darbro, Andrew M. Bellizzi, Michael D. Henry, Dawn E. Quelle

**Affiliations:** 1 Department of Neuroscience and Pharmacology, University of Iowa, 2-570 Bowen Science Building, 51 Newton Road, Iowa City, IA 52242 USA; 2 Cancer Biology Graduate Program, University of Iowa, Iowa City, IA USA; 3 Department of Internal Medicine, University of Michigan, Ann Arbor, MI USA; 4 Holden Comprehensive Cancer Center, University of Iowa, Iowa City, IA USA; 5 Department of Biostatistics, University of Iowa, Iowa City, IA USA; 6 Department of Pediatrics, University of Iowa, Iowa City, IA USA; 7 Department of Pathology, University of Iowa, Iowa City, IA USA; 8 Department of Molecular Physiology and Biophysics, University of Iowa, Iowa City, IA USA; 9 Department of Urology, University of Iowa, Iowa City, IA USA

**Keywords:** Cancer models, Metastasis

## Abstract

Pancreatic neuroendocrine neoplasms (pNENs) are slow growing cancers of increasing incidence that lack effective treatments once they become metastatic. Unfortunately, nearly half of pNEN patients present with metastatic liver tumors at diagnosis and current therapies fail to improve overall survival. Pre-clinical models of pNEN metastasis are needed to advance our understanding of the mechanisms driving the metastatic process and for the development of novel, targeted therapeutic interventions. To model metastatic dissemination of tumor cells, human pNEN cell lines (BON1 and Qgp1) stably expressing firefly luciferase (luc) were generated and introduced into NSG immunodeficient mice by intracardiac (IC) or intravenous (IV) injection. The efficiency, kinetics and distribution of tumor growth was evaluated weekly by non-invasive bioluminescent imaging (BLI). Tumors formed in all animals in both the IC and IV models. Bioluminescent Qgp1.luc cells preferentially metastasized to the liver regardless of delivery route, mimicking the predominant site of pNEN metastasis in patients. By comparison, BON1.luc cells most commonly formed lung tumors following either IV or IC administration and colonized a wider variety of tissues than Qgp1.luc cells. These models provide a unique platform for testing candidate metastasis genes and anti-metastatic therapies for pNENs.

## Introduction

Pancreatic neuroendocrine neoplasms (pNENs) are incurable, uncommon malignancies that are steadily rising in incidence^1^. NENs, in general, are clinically challenging because they are slow growing, biologically diverse tumors that lack effective therapies once they become metastatic. Gastroenteropancreatic NENs often elude diagnosis for years; consequently, ~ 40% of patients or more have liver metastases at the time of diagnosis^1–6^. While considered rare, NENs progress relentlessly and the most frequent types (arising in the pancreas, small bowel and lung) have risen 4- to 6-fold in incidence over the last few decades^1–3^.

Important modalities for treating metastatic NENs include hepatic cytoreduction, peptide receptor radionuclide therapy, somatostatin analogs, select chemo-therapies, and targeted therapies with the mammalian target of rapamycin (mTOR) inhibitor everolimus, and receptor tyrosine kinase inhibitors or sunitinib^5,7–12^. However, none of those approaches are curative as resistance invariably develops, and overall survival is not improved. The situation is worsened by the fact that little is known about key molecular mechanisms driving NEN progression. Recent RNA sequencing studies of metastatic lesions from pNEN patients identified potential metastasis genes^13,14^, but their role in NEN cell migration, invasion and colonization have yet to be tested. Deeper mechanistic insight into the metastatic cascade of NENs and more effective targeted therapies are needed to improve treatment options for patients with advanced NEN disease.

A major barrier to understanding NEN metastasis is the paucity of disease-relevant models for preclinical investigations. There are just three authenticated human pNEN cell lines available for in vitro analyses of cell migration and invasion, including the two most studied lines, BON1 and Qgp1^15^. BON1 was developed in 1991 from a lymph node metastasis from a pNEN patient while Qgp1 was established from a pancreatic islet cell tumor in 1980^16,17^. Both are non-functional (i.e., non-hormone secreting) pNENs, which is similar to the majority of pancreatic NENs that arise in people. In vivo models of pNEN metastasis are also limited. A small number of genetically engineered mice exist that develop spontaneous pNENs but, until recently, none were suitable for studying metastasis due to the absence or low frequency of metastatic tumor formation^18–20^. In 2019, Kobayashi developed RIP-Tag2;AB6F1 mice; these animals express SV40 large T antigen under a rat insulin promoter and form non-functional pNENs that metastasize to the liver^21^. While that model is expected to significantly advance pNET research, it has several inherent limitations. These include variable tumor onset, inability to track tumor progression to internal organ sites without expensive imaging (such as MRI or CT), and the fact that the metastatic phenotype is not fully penetrant (occurs in ~ 65% of the mice)^21^.

In this study, we describe the development and characterization of two reliable methods for modeling pNEN metastasis in vivo. The models employ bioluminescent derivatives of BON1 and Qgp1 pNEN cells that express the bioluminescent protein, firefly luciferase, and are introduced into mice by intracardiac (IC) or intravenous (IV) delivery. This enables real time, non-invasive serial assessment of tumor development over time using bioluminescence imaging (BLI)^22,23^. BLI-based tumor models are advantageous since they provide a sensitive measure of photon emission from luciferase-expressing cancer cells throughout the body, even in deep tissue locations, with signals correlating well and linearly with tumor growth^22,24,25^. While direct administration of cancer cells into the bloodstream bypasses the early steps of metastasis (invasion and intravasation), it effectively mimics the later stages of metastasis (tumor cell dissemination, extravasation and tissue colonization). Here, we demonstrate these models are fast, feasible and reliable systems to study pNEN metastasis, providing a much-needed platform for testing candidate metastasis genes and clinically relevant therapeutics.

## Results

### Development of bioluminescent human pNEN cell lines

**Figure 1:**
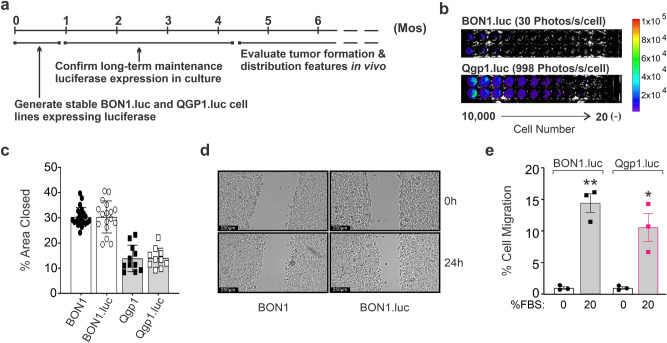
Development of bioluminescent pNEN cells and mouse models of metastasis. (**a**) Timeline showing initial generation of luciferase-expressing cell lines and time required to validate sustained bioluminescence and perform in vivo tumor studies. (**b**) In vitro assay of luciferase activity in serially diluted BON1.luc and Qgp1.luc cells following G418 selection. (**c**) Quantification of scratch assay migration data for parental cells and luciferase-expressing derivatives, as measured by the percent wound closure (% area closed) from 3 or more experiments, each with at least 3 replicates. Percent wound closure was measured using ImageJ version 1.8.0 freely downloaded from NIH (https://imagej.nih.gov/ij/download.html) (**d**) Representative images of the scratch assay in BON1 and BON1.luc cells at 0 and 24 h. (**e**) Transwell migration assay showing the fold increase in migration stimulated by 20% FBS relative to 0% FBS control. Samples were plated in triplicate and results replicated in at least three independent experiments. Representative data from one experiment are shown as mean ± SD; *, *p* = 0.05; **, *p* = 0.01; Welch t-tests with unequal variance comparing results between 0 and 20% FBS for each cell type.

We sought to generate novel mouse models of pNEN metastasis in which tumors would develop rapidly, reproducibly and could be tracked in real time through non-invasive BLI. The overall goals and timeline of this study are outlined in Fig. 1a. To generate stable pNEN cell lines expressing luciferase, parental BON1 and Qgp1 pNEN cells were transfected with a luciferase expression vector containing a G418 (*neomycin*) resistance gene^22^. Successfully transfected cells were selected for 2 weeks in G418 containing media and the resulting polyclonal populations were tested for bioluminescence using an in vitro luciferase activity assay (Fig. 1b). BON1.luc cells displayed relatively low luciferase activity (~ 30 photons/sec/cell) whereas Qgp1.luc cells showed much higher bioluminescent intensity (998 photons/s/cell). Similar ranges in bioluminescence have been seen in other cells with 20–30 photons/s/cell for several pancreatic ductal adenocarcinoma cell lines^26^ and 250–700 photons/s/cell for PC-3 and 22Rv1 prostate cancer cells^22,27^, likely reflecting differences in the sites and frequency of stable integration by the luciferase expression construct. Both cell lines were then cultured without antibiotic for at least 4 weeks to confirm stable maintenance of luciferase activity in the absence of selection (Fig. 1a), a pre-requisite for tracking in vivo tumor formation and metastasis by BLI.

Prior studies demonstrated in vitro migratory activity of BON1 and Qgp1 cells using transwell (Boyden chamber) and scratch (wound healing) migration assays^28,29^. Scratch assays were performed to compare the in vitro migration of the luciferase-expressing derivatives to their non-bioluminescent parental counterparts. No differences in migratory capacity were observed (Fig. 1c,d). We then assessed the migration potential of the newly generated BON1.luc and Qgp1.luc populations in a standard transwell assay (Fig. 1e). Both cell lines displayed significant migration through the membrane following overnight exposure to 20% FBS relative to negative controls exposed to 0% FBS.

### Faster rates of tumor formation for Qgp1.luc cells in the intracardiac (IC) model

Each bioluminescent pNEN cell line was then delivered into the arterial circulation of immunodeficient Nod-Scid-Gamma (NSG) mice through direct injections into the left ventricle, thereby enabling hematogenous dissemination of the cells^30^. Using this intracardiac (IC) approach, all animals (n = 16, 8 for each cell line) survived the injection process and 100% formed tumors.

**Figure 2:**
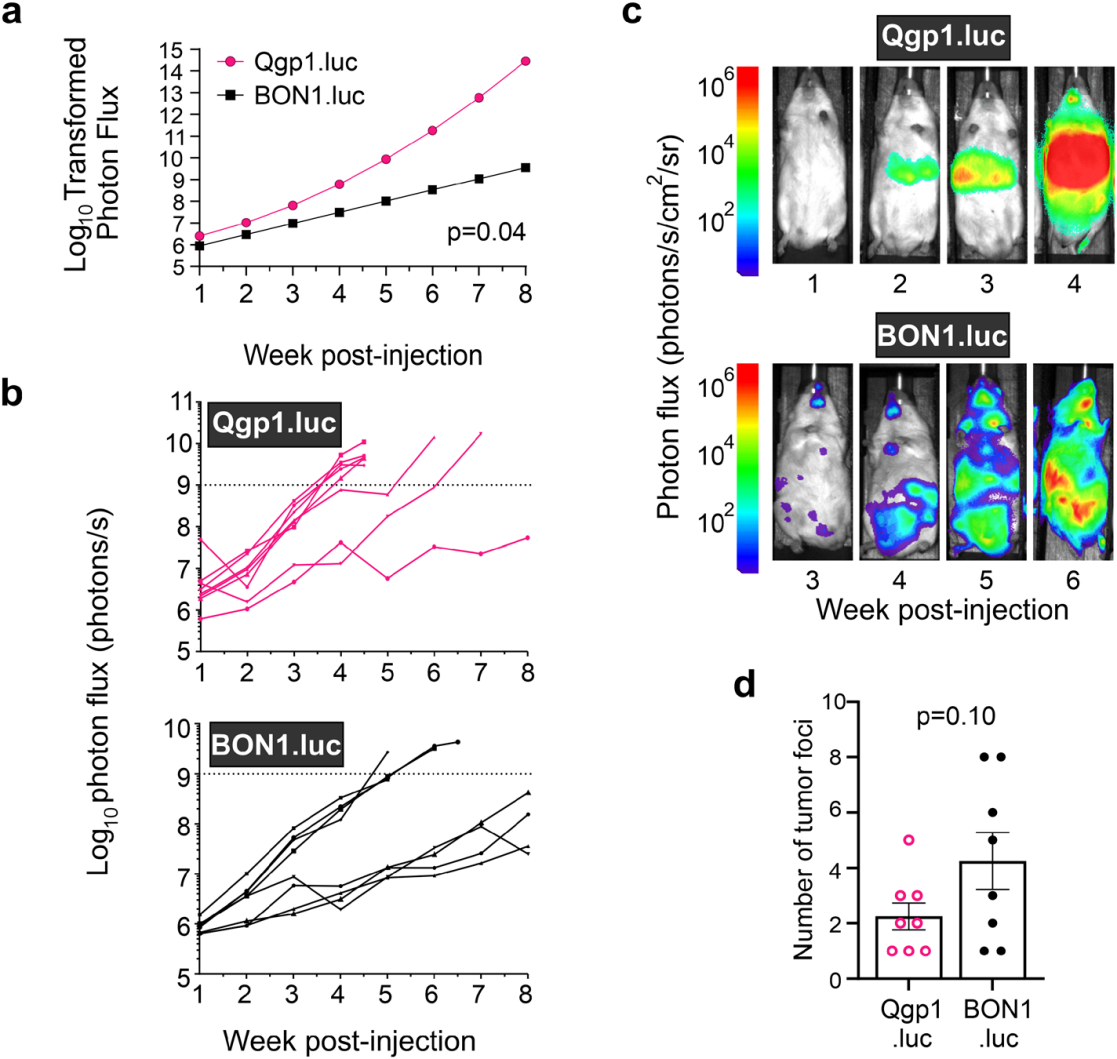
Intracardiac model of pNEN metastasis. (**a**) Average tumor growth rates as measured by Log_10_ transformed photon flux in Qgp1.luc and BON1.luc injected mice. *p* = 0.04 was obtained from linear mixed effects regression models on data for all mice (shown in b) to estimate and compare tumor growth curves. Qgp1.luc tumors grew faster than BON1.luc tumors. (**b**) Graphs showing individual tumor growth rates in NSG mice (n = 8 per group), as measured by BLI, following intracardiac injection with Qgp1.luc (top) or BON1.luc (bottom) cells. Each line represents a single mouse with tumor growth quantified by total photon flux (photons per second) per animal over the indicated time. (**c**) Longitudinal bioluminescence images at the indicated times (weeks) post-injection with Qgp1.luc (top) or BON1.luc (bottom) cells. Each set of images was taken from the same mouse. (**d**) Average number of tumor foci per mouse in BON1.luc versus Qgp1.luc animals based on quantified photon flux of tumors by ex vivo BLI. *p* = 0.10 by Students t-tests with equal variance comparing the number of tumors between cell types.

The two cell lines displayed variable performance in the IC model. Qgp1.luc cells were generally more aggressive with a significantly faster rate of tumor growth than BON1.luc (Fig. 2a,b). Quantification of whole-body tumor growth by weekly BLI of each animal showed that maximal tumor burden (approximately 10^9^ photons/s) was reached by 4 weeks for the majority of Qgp1.luc mice (Fig. 2b). By comparison, half of the BON1.luc tumors grew to maximal size by 5 weeks post-injection while the other half progressed more slowly and only reached an average of ~ 10^9^ photons/s by 8 weeks, the pre-determined endpoint (used instead of death) for the study. Prior BLI tumor studies have shown that this value, not a volume measurement but instead based on total non-saturated bioluminescence signal, correlates with significant tumor growth and the onset of reduced health in the mice. Kaplan–Meier analysis of survival using the log rank test for group comparisons revealed a marginally significant difference in overall survival (*p* = 0.06). Median overall survival for Qgp1.luc was 4 weeks versus 7 weeks for BON1.luc injected mice.

Serial bioluminescence images in vivo revealed discrete tumor foci had formed by 2–3 weeks for Qgp1.luc whereas BON1.luc tumors became detectable 4–5 weeks after injection (Fig. 2c). Accurate quantification of individual lesion bioluminescence by in vivo imaging is challenging. Therefore, ex vivo imaging of the mice was performed when maximal tumor burden was reached to accurately quantify the average number of tumor foci in each IC model. A trend of fewer tumors per each Qgp1.luc mouse compared to BON1.luc animals was observed although this was not statistically significant (Fig. 2d, *p* = 0.10). On average, Qgp1.luc mice had 2 tumors per animal while BON1.luc formed 4 tumors per mouse.

### Preferential metastasis to the liver for Qgp1.luc cells in the IC model

**Figure 3:**
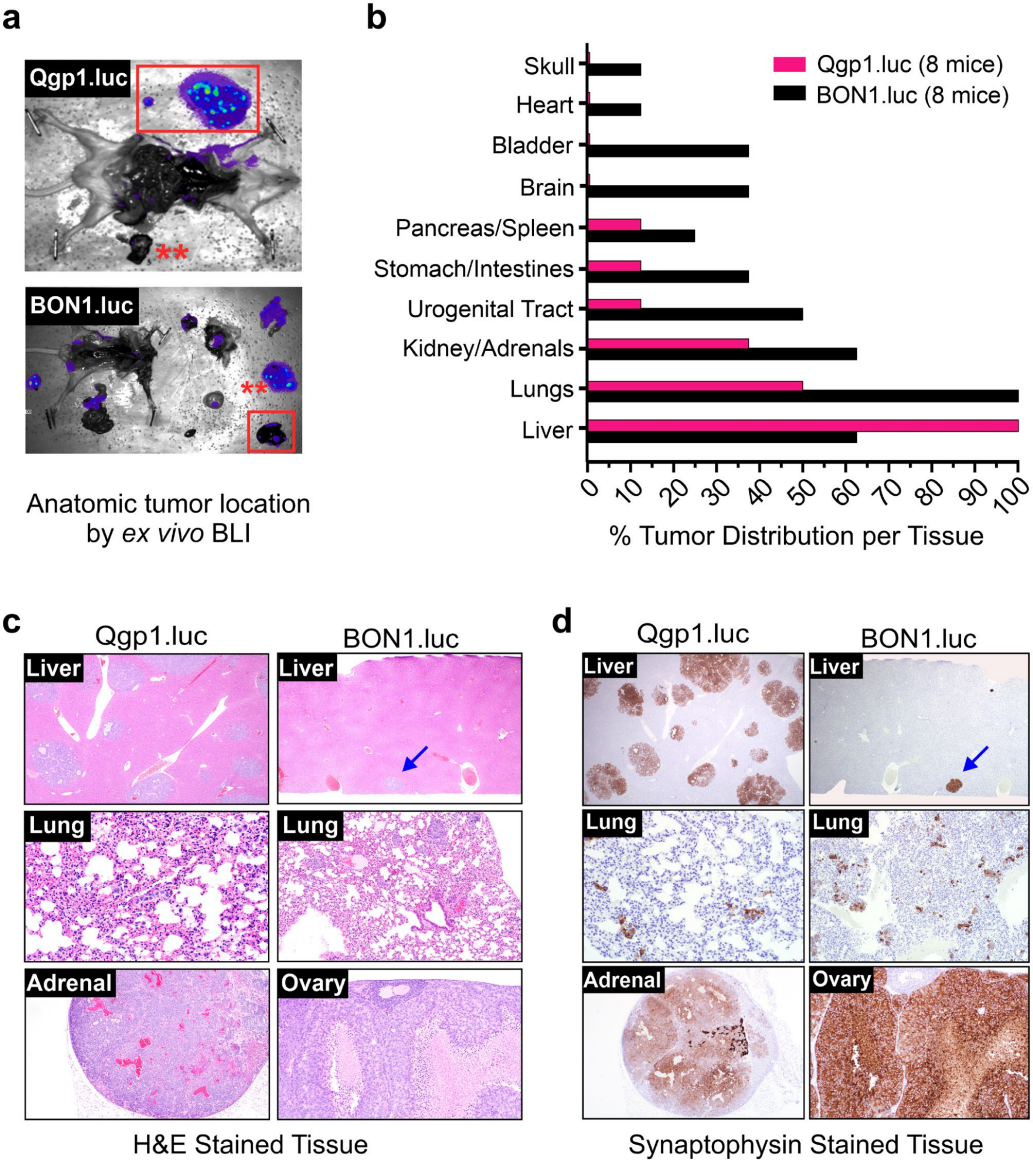
Sites and frequency of tumor distribution in the IC model. (**a**) Ex vivo bioluminescent images (BLI) of tissues to pinpoint the different organ sites of tumor cell colonization. Red boxes, liver; red asterisks, lung. (**b**) Quantified distribution of bioluminescent pNEN cells per tissue. All BON1.luc-injected mice exhibited lung tumors while all Qgp1.luc-injected mice exhibited liver tumors. (**c**) H&E images of Qgp1.luc and BON1.luc tumors in the liver, lung, adrenal and ovary tissues, as indicated in the inset of each image. Tumor cells are stained purple with discrete lesions (upper panels), scattered individual cells throughout the tissue (middle panels), or large lesions overtaking the normal tissue (bottom panels) shown. Blue arrow (top right panel) identifies a single, small BON1.luc liver tumor. (**d**) IHC staining (brown) for the NEN marker, synaptophysin, on the same tissues shown in (c).

In vivo BLI enables non-invasive tumor tracking but is unable to determine the exact locations where tumors develop. Therefore, ex vivo imaging of the mice (when maximal tumor burden was reached) was also used to pinpoint the precise anatomic locations and distribution frequency of the tumors (Fig. 3a). Consistent with BLI tumor data in Fig. 2, Qgp1.luc formed lesions at fewer organ sites and the liver was the major site of metastasis with 100% (8 out of 8) animals developing liver tumors (Fig. 3a,b). Other common sites of Qgp1.luc metastasis were the lungs (50%) and kidneys or adrenal glands (35%) (Fig. 3b). In contrast, BON1.luc tumors appeared in a wider range of tissues. Lung metastases were seen in 100% of the BON1.luc mice while the liver, kidneys/adrenals and urogenital tract were colonized in at least half of the animals (Fig. 3b). BON1.luc tumors also developed frequently in the bladder, brain and stomach/intestines (~ 35%) and to a lesser degree at several other sites including the heart and skull (in the bone).

Histologic analyses confirmed the mouse organ that was colonized (Fig. 3c). In general, Qgp1.luc tumors in the liver were multi-focal with a satellite pattern often seen in NEN patients whereas BON1.luc liver tumors were small and few in number (Fig. 3c, top panels). Lung tumors for BON1.luc were also relatively small but distinct while Qgp1.luc typically formed microscopic (individual cell) metastases in the lung (Fig. 3c, middle panels). Tumor cells were verified by immunohistochemical staining for synaptophysin, a NEN marker (Fig. 3d). Both cell types formed metastatic lesions in the adrenal glands (less often in the kidney), as shown for the large macroscopic adrenal tumor formed by Qgp1.luc cells (Fig. 3c, lower left panel). While not a typical site of NEN metastasis, adrenal metastases do occasionally present clinically in NEN patients^31,32^. Metastatic colonization of the ovary, commonly seen in patients with midgut NENs, was frequent in this model for BON1.luc cells with tumors often becoming quite large and taking over the normal tissue (Fig. 3c, lower right panel). Altogether, Qgp1.luc tumors had a more restricted distribution with preferential colonization of the liver whereas BON1.luc cells consistently metastasized to the lung in addition to a wider array of tissues.

### Differential tumor growth rates for bioluminescent pNEN cells in the intravenous (IV) model

Qgp1.luc and BON1.luc cells were also introduced into NSG mice through intravenous (IV, lateral tail vein) injection. This procedure is simpler than the IC injection approach described above. BLI performed immediately post-injection demonstrated similar uptake of the bioluminescent cells for all mice, and 100% of the mice survived the IV delivery. All animals formed tumors.

**Figure 4:**
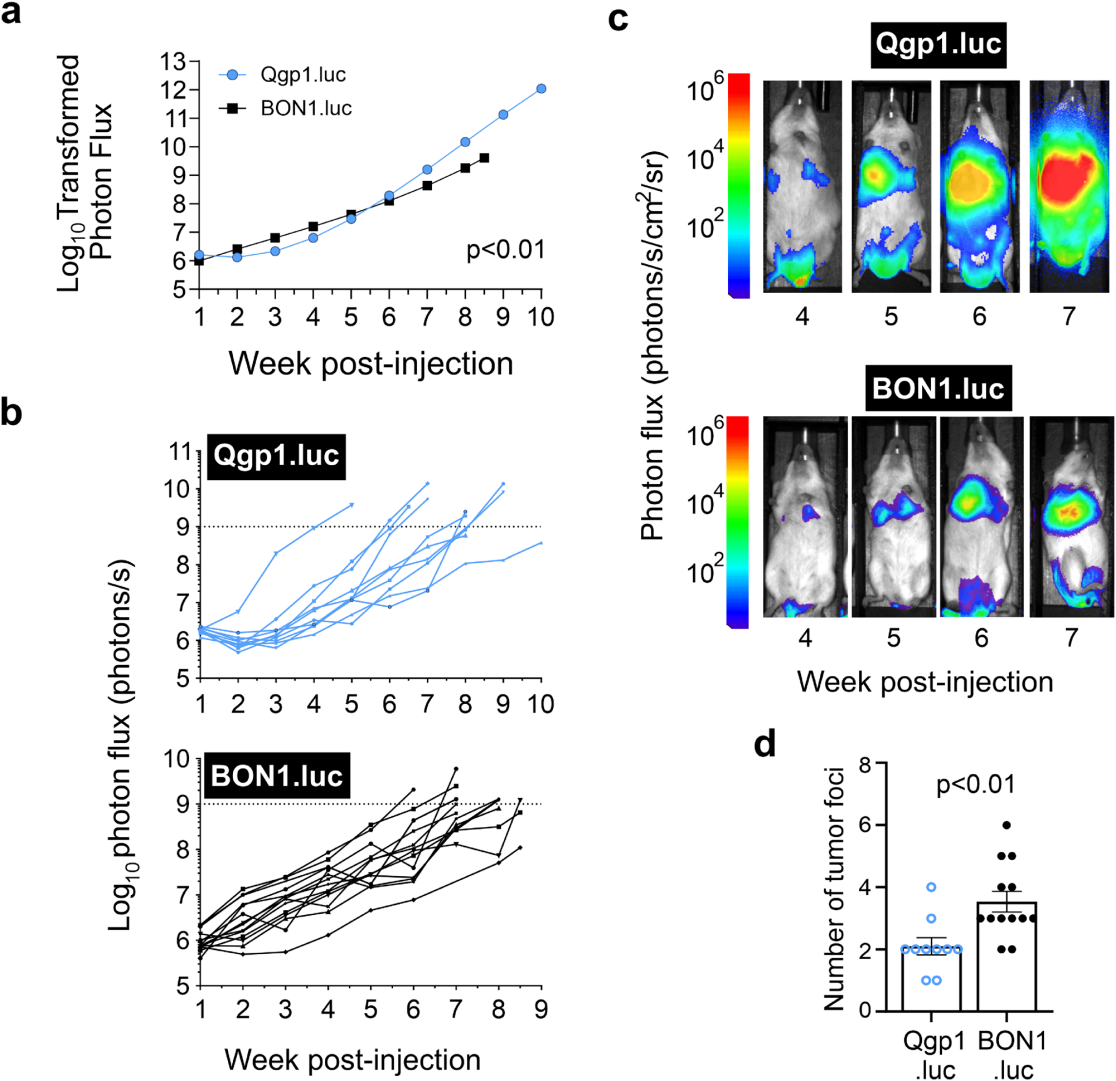
Intravenous model of pNEN metastasis. (**a**) Average tumor growth rates as measured by log-transformed photon flux in Qgp1.luc and BON1.luc injected mice. *p* < 0.01 was obtained from linear mixed effects regression models on data for all mice (shown in **b**) to estimate and compare tumor growth curves. (**b**) Graphs showing individual tumor growth rates, as measured by in vivo BLI, in each NSG mouse following intravenous injection of Qgp1.luc cells (top, n = 10 mice) and BON1.luc cells (bottom, n = 13 mice). Each line reflects a single mouse with tumor growth quantified by total photon flux (photons per second) over the indicated time. (**c**) Longitudinal bioluminescence images at the indicated times in weeks post-injection with Qgp1.luc (top) and BON1.luc (bottom) cells. Each set of images was taken from the same mouse. (**d**) Average number of tumor foci per mouse in Qgp1.luc and BON1.luc animals based on quantified photon flux of tumors by ex vivo BLI. *, *p* < 0.01 by Students t-tests with equal variance comparing the number of tumors between cell types.

A significant difference in tumor growth rates was observed between the cell types following IV injection (Fig. 4a). Qgp1.luc had lower tumor burden than BON1.luc until approximately 5 weeks post-injection at which time Qgp1.luc surpassed BON1.luc. Maximal tumor burden was reached at similar times, by approximately 7–9 weeks, for most Qgp1.luc and BON1.luc mice (Fig. 4b). Serial bioluminescence images from each mouse revealed easily detectable tumor foci by 4–5 weeks post-injection for both cell lines (Fig. 4c). Similar to results in the IC model, ex vivo imaging of the mice (performed at maximal tumor burden) showed that Qgp1.luc mice formed fewer tumors compared to BON1.luc tumors (Fig. 4d, *p* < 0.01). While Qgp1.luc mice had an average of 2 lesions formed per mouse, BON1.luc mice formed approximately 3.5 tumors per mouse.

### Qgp1.luc cells preferentially metastasize to the liver in the IV model

**Figure 5:**
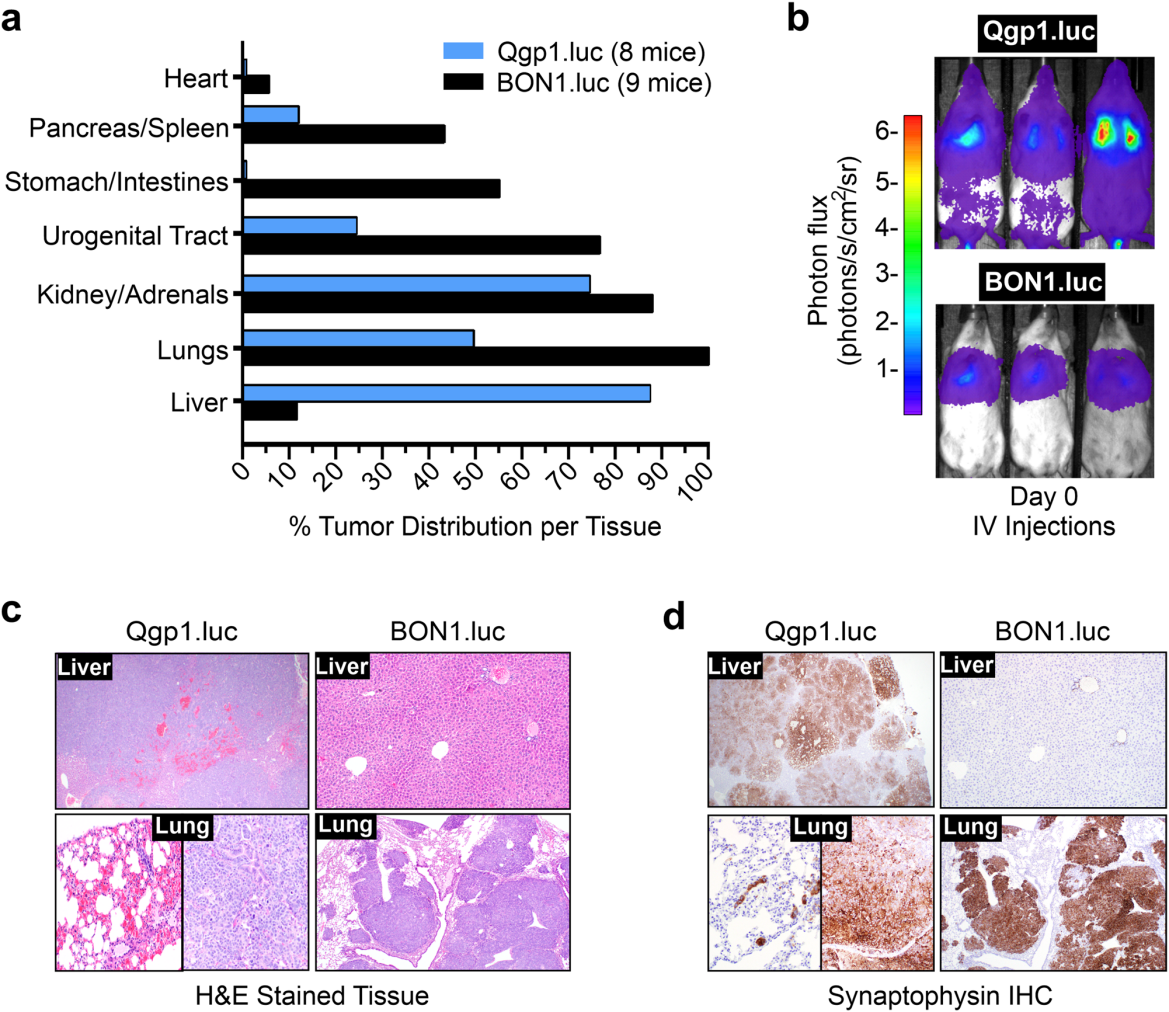
Sites and frequency of tumor distribution in the IV model. (**a**) Quantified distribution of bioluminescent pNEN cells per tissue by ex vivo BLI. All BON1.luc-injected mice (9 out of 9) exhibited lung tumors while most QGP1.luc-injected mice (7 of 8) exhibited liver tumors. (**b**) In vivo BLI of mice immediately following injection with Qgp1.luc and BON1.luc cells (Day 0). Three representative mice in each group are shown. (**c**) Representative H&E images of Qgp1.luc and BON1.luc tumors that colonized the liver and lung, as indicated. The liver in a representative BON1.luc mouse shows no tumors in that tissue. (**d**) IHC staining (brown) for the NEN marker, synaptophysin, on the same tissues shown in (**c**).

Ex vivo imaging at maximal tumor burden was once again employed to pinpoint the precise organ locations and distribution frequency of the tumors (Fig. 5a). Similar to the IC model of metastasis, Qgp1.luc lesions formed lesions at fewer organ sites with the liver presenting as the major site of metastasis. Specifically, ~ 90% of Qgp1.luc animals (7 out of 8 mice imaged) developed liver tumors (Fig. 5a). Other common sites of Qgp1.luc metastasis were the kidneys and/or adrenal glands (75%) while half of the mice formed lung tumors. The latter result of only 50% lung tumors was unexpectedly low given that the lung capillary beds are the initial site of tumor cell delivery following IV injections of tumor cells^33^. Importantly, we verified that all Qgp1.luc and BON1.luc injected mice displayed localized, bioluminescent signals in the lungs immediately following IV injections (Fig. 5b).

BON1.luc tumors grew in most of the same tissues as Qgp1.luc tumors as well as additional sites not colonized by the other cell line, namely the stomach/intestines and heart. Notably, BON1.luc displayed a strong preference for colonizing the lungs (100%) as well as the kidneys/adrenals (88%), urogenital tract (76%) and stomach/intestines (60%).

Confirmation of human NEN origin and type of tissue colonized was obtained by histologic analyses of the lesions. The top panels of Fig. 5c show typical H&E images of the livers from IV injected mice. Qgp1.luc often formed extensive liver tumors that took over the normal tissue. By comparison, normal livers were observed in most of the BON1.luc mice (top right image), reflecting the fact that only a single BON1.luc IV mouse (1 of 9, 11%) formed tumors at that site. The absence of liver tumors in nearly all BON1.luc animals was verified by IHC for the NEN marker, synaptophysin, which was negative in the livers of all but one animal (Fig. 5d). In contrast, BON1.luc frequently formed large and multi-nodal tumors in the lungs (lower right panel). The morphology of Qgp1.luc lung tumors was varied with some mice forming micro-metastases (bottom left panel) and other animals bearing large tumors (bottom middle panel). To sum up the IV model, Qgp1.luc tumors grew preferentially in the liver while BON1.luc colonized a wider range of tissues with highest frequency in the lungs, kidney/adrenals, and urogenital tract.

## Discussion

This study sought to establish xenograft mouse models of pNEN metastatic colonization that may be used for rapid and relatively inexpensive pre-clinical assessment of candidate NEN metastasis genes and therapies. There is a great need for such models in the NEN field where ~ 40% of patients are diagnosed with distant metastases and few genetic models of NEN metastasis exist. Here, we developed two reliable methods for modeling NEN metastatic colonization in mice. The models employ newly generated bioluminescent pNEN cell lines which displayed significant migratory activity in vitro and successfully formed metastatic tumors in vivo. To facilitate comparison of the tumor distribution and frequency in the different models, results are summarized in Table 1. Importantly, whole body tumor growth can be quantitatively tracked over time through non-invasive imaging while ex vivo imaging must be used to verify precise tissue locations of the tumors. Tumor monitoring via real-time BLI measurements reduces the number of animals required for analyses.

**Table 1:** Summary of BLI model findings.

Tumors	Qgp1.luc		BON1.luc	
	IC	IV	IC	IV
# per mouse	2.3 ± 0.49	2.1 ± 0.28	4.3 ± 1.03	3.5 ± 0.33
Liver	100%	87.5%	62.5%	11.1%
Lungs	50%	50%	100%	100%
Kidney/adrenals	37.5%	75%	62.5%	88.9%
Urogenital tract	12.5%	25%	50%	77.8%
Stomach/intestines	12.5%	0%	37.5%	55.6%
Pancreas/spleen	12.5%	12.5%	25%	44.4%
Brain	0%	0%	37.5%	0%
Skull	0%	0%	12.5%	0%
Heart	0%	0%	0%	11.1%
Unknown abdominal*	0%	12.5%	0%	22.2%

*Precise anatomic location inadvertently hindered during necropsy.

We evaluated the efficiency of tumor formation and patterns of tumor distribution following IC or IV administration of the two bioluminescent pNEN cell lines. Both delivery approaches were highly efficient, producing tumors in all injected animals. The IC method delivers cancer cells directly into the arterial circulation and yielded a slightly broader tissue distribution of tumors, at least for BON1.luc cells. The high rate of liver tumor formation for both cell lines (100% for Qgp1.luc and > 60% for BON1.luc) following IC injection is clinically relevant since the liver is the most common site of metastasis in advanced NEN patients and it worsens prognosis^1,3,4,34^. IC delivery may also be useful for exploring atypical sites of NEN metastasis linked with poor outcomes, such as the brain^35–37^, as this organ was colonized by BON1.luc cells in nearly 40% of IC mice. The main challenge with the IC model is perfecting the delivery of cells into the left ventricle, which requires significant experience.

The IV model employs a comparatively easier method of tumor cell delivery. The simplicity of that approach may expand the number of animals that can be tested and facilitating its use by more investigators. Interestingly, while BON1.luc cells almost always formed lung tumors regardless of the delivery method, remarkably, Qgp1.luc cells preferentially metastasized to the liver in both IC and IV models. This is evident in spite of the fact that Qgp1.luc cells are 30-fold brighter than BON1.luc cells and therefore allow for more sensitive detection. Although it is possible that intense signal from the liver in Qgp.1luc injected mice obscures weaker signals at other organ sites in vivo, ex vivo imaging with the liver removed was also consistent with more liver-specific colonization of Qgp1.luc cells.

Preferential colonization of the liver following IV injection of Qgp1.luc cells is surprising since cancer cells introduced into the tail vein initially pass through the lung capillary beds, typically resulting in lung tumor formation^33^. Yet only 50% of Qgp1.luc IV mice formed lung tumors. It is possible the liver provides a more suitable microenvironment than the lung or other tissues to support Qgp1 colonization, therefore this cell line may afford an experimental platform to explore liver tropism of pNEN metastasis. In pNEN patients, metastasis to the liver occurs via the splanchnic circulation, resulting in a higher exposure of the liver to metastatic cells. Animal models of liver metastasis often employ surgically involved portal vein or intrasplenic injections. However, here we show that the comparatively simpler IC and IV injections with Qgp1.luc cells result in liver metastases with high efficiency. Notably, both IC and IV approaches circumvent the early steps of metastasis, invasion and intravasation, although the models recapitulate tumor cell dissemination through the bloodstream, blood vessel extravasation, and seeding / colonization of tissues. These approaches have been used with success to model metastasis of many other tumor types including pancreatic ductal adenocarcinoma, retinoblastoma, breast cancer, and prostate cancer^22,26,38–40^.

The development, careful evaluation and comparison of these IC and IV pNEN metastasis models is a first in the NEN field. The models will enable rapid testing of innovative therapies with potential anti-metastatic activity, whether it be assessing the efficacy of individual drugs or unique combinations that display synergistic anti-tumor activities in vitro and in other systems. In particular, these models should provide a valuable setting to explore the anti-metastatic activities of peptide radioligand receptor therapies (PRRT), a mainstay for pNEN treatment, paired with targeted inhibitors of pro-metastatic pathways. The bioluminescent pNEN cells can also be manipulated genetically to express altered levels or mutant forms of candidate metastasis genes to examine their in vivo role in pNEN metastasis.

Qgp1 and BON1 cells are non-functional pNEN lines, reflecting the vast majority of patient pNENs. This makes them valuable NEN research tools as they are the only non-functional, human pNEN lines available. Moreover, both lines retain wild-type retinoblastoma (RB1) tumor suppressor expression^41^, a feature of low-grade pNENs that is lost in high-grade tumors. Nonetheless, it would be valuable to develop similar pNEN metastasis models using functional pNEN cell lines as they represent up to 30% of pancreatic NENs. Two human insulinoma lines recently became available, NT-3 cells^42^ and the first patient-derived pNEN xenograft, also an insulin-secreting islet tumor^43^. As a well-differentiated, slow growing pNEN with moderate Ki-67 at ~ 15% (unlike BON1 and Qgp1 cells which display high-grade Ki-67 positivity at ~ 80% and rapid proliferation)^42^, bioluminescent NT-3 cells would provide a unique model of low-grade pNEN metastasis.

In summary, current treatments for metastatic NEN disease have limited efficacy and fail to improve overall survival. A better understanding of key drivers of the metastatic process and improved therapeutic options are urgently needed. This work provides a powerful platform for conducting pre-clinical studies of putative NEN metastasis genes and promising therapeutics.

## Materials and methods

### Cell culture

BON1 cells were maintained in Gibco Dulbecco's Modified Eagle Medium Nutrient Mixture F-12 (DMEM/F-12) containing 10% heat inactivated FBS, 2% L-glutamine and 1% penicillin/streptomycin. These cells were originally developed and authenticated by Dr. Courtney Townsend (University of Texas Medical Branch, Galveston, TX)^16^. Qgp1 cells were maintained in Gibco RPMI 1640 Media containing 10% heat inactivated FBS, 1% L-glutamine and 1% penicillin/streptomycin. These cells were purchased from the Japanese Collection of Research Bioresources (JCRB0183). Both lines were maintained at low passage from thaw, routinely tested for mycoplasma contamination and found to be negative, and have been more recently authenticated by immunophenotyping, copy number profiling and whole-exome sequencing^15^.

### Development of luciferase cell lines

BON1 and Qgp1 cells were nucleofected with PGL3 luciferase expression vector using Nucleofector II device (Amaxa biosystems) and selected for at least 2 weeks with 0.75 mg/mL geneticin (G418) to generate stable lines expressing luciferase. Stable lines were maintained in media containing 0.4 mg/mL G418. An in vitro luciferase activity assay was performed to assess the bioluminescent activity of BON1.luc and Qgp1.luc cells. A standard curve was performed by serial dilution of cells (10,000 to 20 cells) in a 96-well black bottomed plate and exposure to 0.15 mg/mL D-luciferin, potassium salt (Gold Bio, cat no: LUCK-100) for 5 min before bioluminescent imaging. The number of photons emitted per second over a 5 min exposure period was measured using an AMI HTX imaging system (Spectral Instruments Imaging).

### Intracardiac injections

All procedures involving animals were conducted according to The University of Iowa Animal Care and Use Committee policies (protocol # 8111590) and ARRIVE guidelines. Intracardiac injections and subsequent analyses were conducted in 6- to 8-week-old male and female immunodeficient NOD-*scid* gamma (NSG) mice (Jackson Laboratories, No. 005557). Mice were anesthetized in a chamber using 2.5% isoflurane, then placed in a ventral position with nose cone anesthesia providing continuous 2.5% isoflurane during the procedure. The chest of each mouse was wiped with 70% ethanol before 100 μl cell suspension (1 × 10^5^ cells) was slowly delivered into the left ventricle of the heart using a 30-gauge needle. To confirm successful delivery following injection, animals were injected intraperitoneally with 200 μL of 15 mg/mL D-luciferin, incubated for 5 min, and imaged on an AMI HTX imaging system (Spectral Instruments Imaging).

### Intravenous tail vein injections

Intravenous tail vein injections and subsequent analyses were conducted on 6- to 8-week-old male and female immunodeficient NOD-*scid* gamma (NSG) mice (Jackson 005557). Mice were placed in a Mouse Tail Illuminator Restrainer (Braintree Scientific) and restrained with tail on platform. Tails were wiped once with 70% ethanol then 200ul (2 × 10^5^ cells) of each cell suspension was delivered through lateral tail vein using a 30-gauge needle. To examine cell distribution following injection, animals were injected intraperitoneally with 200 ul of 15 mg/ml D-luciferin, incubated for 5 min then imaged using AMI HTX BLI system (Spectral Instruments Imaging).

### Bioluminescence imagining analysis of tumor growth and distribution

Metastatic tumor formation and colonization were monitored weekly using an AMI HTX imager (Spectral Instruments Imaging, Tuscon AZ). Mice were anesthetized with 2.5% isoflurane in a chamber, moved to nose cone and maintained anesthesia at 2.5% isoflurane during imaging. Mice were injected intraperitoneally with 200 µl of 15 mg/ml D-luciferin substrate, incubated 5 min then dorsal and ventral images were taken.

### In vitro cell migration assays

For the transwell assay, adherent BON1.luc and Qgp1.luc cells were washed four times with PBS and serum starved for 48 h in serum-free DMEM containing 1% bovine serum albumin (BSA) prior to assaying migration. Cells were then trypsinized and plated (5 × 10^4^ in 100 μl serum-free DMEM or RPMI media) into fibronectin-coated transwell inserts and incubated 10 min to allow cells to settle. To coat the permeable transwell inserts (Corning No. 3422: 24-well, 6.5 mm pore size, 8 micro-pore polycarbonate membrane), inserts were incubated for 30 min at 37 °C with fibronectin (1 mg/ml, 100 μl) and the solution aspirated before plating the cells. The lower chambers beneath each insert were filled with 600 µl of DMEM or RPMI media containing either 0% FBS or 20% FBS as chemoattractant. Plates were then incubated for 24 h at 37 °C, 5% CO_2_ for 24 h to allow migration. Non-migrated cells on top of the inserts were wiped away with a sterile cotton swab. The percent of migrated cells on the underside of each transwell membrane was quantified by BLI using the AMI HTX system following incubation for 5 min with 300 μl of D-luciferin (0.15 mg/ml final concentration). All assays were performed in triplicate and replicated in at least 3 separate experiments. Data were presented as the mean ± SEM and subjected to t-tests with unequal variance to assess the significance of the results.

For the scratch (wound healing) assay, parental BON1 or Qgp1 cells were plated in 12-well dishes along with corresponding BON1.luc or Qgp1.luc cell lines. Cells were allowed to grow to a confluent monolayer then treated with 5ug/ml mitomycin C for 90 min. Cells were washed with several times with PBS, then scratched with a sterile p200 tip and placed in DMEM with 0.1% FBS. Scratch locations were denoted with a permanent marker on each well and wounds were imaged immediately and again at 24 h. Total area of each wound at the different time points was measured using ImageJ software version 1.8.0 freely downloaded from NIH (https://imagej.nih.gov/ij/download.html) and percent wound closure was calculated.

### Data availability

The bioluminescent pNEN cell lines, detailed procedures and data obtained in this study are available from the corresponding author upon request.

### Statistical analysis

T-tests were used for the comparison of cell migration and number of tumor foci. Linear mixed effects regression models were used to estimate and compare tumor growth curves between pNET cell lines, BON1.luc and Qgp1.luc. The Kaplan–Meier method was used to estimate survival curves and cell line comparisons were made using the log-rank test. If a mouse did not meet criteria for survival and was euthanized for other reasons (e.g., low body conditioning score or poor mobility), the mouse was treated as a censored observation. All tests were two-sided and assessed for significance at the 5% level using SAS v9.4 (SAS Institute, Cary, NC).

### Ethical approval

All mouse experiments were approved by the Institutional Animal Care and Use Committee (IACUC) at the University of Iowa and were performed according to the NIH animal use guidelines.
